# Potential cellular receptors involved in hepatitis C virus entry into cells

**DOI:** 10.1186/1476-511X-4-9

**Published:** 2005-04-19

**Authors:** Daniel Favre, Beat Muellhaupt

**Affiliations:** 1 Division of Gastroenterology and Hepatology, Department of Internal Medicine, University Hospital Zürich, Rämistrasse 100, CH-8091 Zürich, Switzerland

**Keywords:** hepatitis C virus, HCV, low density lipoproteins, LDL, CD81, SR-B1, exosomes, receptor, dextran sulfate, infection, lipids, statins

## Abstract

Hepatitis C virus (HCV) infects hepatocytes and leads to permanent, severe liver damage. Since the genomic sequence of HCV was determined, progress has been made towards understanding the functions of the HCV-encoded proteins and identifying the cellular receptor(s) responsible for adsorption and penetration of the virus particle into the target cells. Several cellular receptors for HCV have been proposed, all of which are associated with lipid and lipoprotein metabolism. This article reviews the cellular receptors for HCV and suggests a general model for HCV entry into cells, in which lipoproteins play a crucial role.

## Review

### Hepatitis C virus (HCV) and cellular receptors for HCV

Hepatitis C virus (HCV) is a major cause of chronic liver disease, with approximately 170 million people infected worldwide [1]. Infection with HCV can lead to hepatocellular carcinoma [2]. To study the adsorption, penetration and replication of the virus, a major obstacle has been the lack of an efficient and reproducible in vitro infection system. Thus, the identification of the HCV receptor on the surface of susceptible cells, especially hepatocytes, remains a major challenge for the development of both in vitro cell culture systems, and for the design of successful therapies [3,4].

Several cellular receptors have been proposed to mediate the entry of HCV into cells, namely the CD81 receptor [5,6], the scavenger receptor class B type I (SR-BI) receptor [7], and the low density lipoprotein (LDL) receptor [8,9].

### CD81 as a receptor for HCV

The tetraspanin CD81 (also named TAPA-1) is a widely-expressed cell surface protein of 26 kDa that is involved in pleiotropic activities such as cell adhesion, motility, metastasis, cell activation and signal transduction [10]. It physically associates with a variety of other membrane proteins such as integrins, lineage-specific molecules and other tetraspanins. It is expressed in most human tissues with the exception of the red blood cells and platelets. Association of CD81 with other molecules has been extensively studied with B and T cells.

It was shown that the expression of CD81 on nonpermissive human, but not murine, hepatic cells enabled the entry of HCV pseudoviruses. The inhibition of viral entry, achieved by application of anti-CD81 monoclonal antibodies, occurred at a step following viral attachment to target cells [11]. When the HCV envelope glycoproteins E1 and E2 were expressed in a baculovirus system, the purified E1-E2 heterodimer interacted with CD81, as well as with the LDL receptor [12]. The human CD81 protein was expressed in bacteria, and the critical amino acids in CD81 involved in the interaction with the viral envelope protein E2 were identified [13]. HIV-HCV pseudotypes bearing native HCV glycoproteins were infectious to the human hepatoma cell lines Huh7 and PLC/PR5. The infectivity was inhibited by recombinant soluble CD81, suggesting that CD81 was a component of the receptor complex [14]. CD81 chimeras, but not wild-type CD81, could internalize recombinant E2 protein and E2-enveloped viral particles from the serum of HCV-infected patients into Huh7 hepatoma cells [15]. Moreover, the expression of CD81 in human liver-derived cells, HepG2 and HH29, that were previously resistant to infection conferred permissiveness to HCV pseudotype infection [16].

In contrast, in several studies, no correlation between CD81 expression and HCV binding has been observed. It has been suggested that HCV binding to hepatocytes might not entirely depend on CD81 [17]. Instead, these authors proposed that CD81 may be an attachment receptor with poor capacity to mediate the viral entry, and that reducing environments may not not favor CD81-HCV interaction. Indeed, it was shown that the binding of E2 to CD81 was not predictive of an infection-producing interaction between HCV and host cells [18]. Moreover, the binding of HCV-like particles was CD81-independent and did not correlate with the expression of the LDL receptor [19]. Finally, human CD81 transgenic mice that lacked expression of the endogenous mouse CD81 were resistant to HCV infection [20]. These authors concluded that the expression of human CD81 alone was not sufficient to confer susceptibility to HCV infection in the mouse.

### SR-BI as a receptor for HCV

Scavenger receptors are cell membrane proteins that bind chemically modified lipoproteins, such as acetylated and oxidized LDLs. These receptors have been categorized into broad classes (A, B, C, D, etc), according to their structures [21]. The SR-BI receptor is involved in bidirectional cholesterol transport at the cell membrane and can bind both high density lipoproteins (HDL) and low density lipoproteins [22]. The cholesterol uptake is different from the classic LDL receptor-mediated endocytosis pathway, since it appears to involve initial transfer to the plasma membrane [23]. SR-BI is highly expressed in hepatocytes [24], and is located in the cholesterol-rich lipid raft membrane compartment [25]. The HCV E2 protein could bind to hepatoma cell lines independently of the CD81 receptor. SR-BI was identified as a mediator of this binding. This interaction was selective, since neither the mouse SR-BI nor the closely related human scavenger receptor CD36, were able to bind E2. The E2 recognition by SR-BI was competitively inhibited, in an isolate-specific manner, by a monoclonal antibody raised against the hypervariable region 1 (HVR1, a 27 amino acid segment located at the N-terminus of the E2 poplypeptide) [7].

### LDL receptor as a receptor for HCV

The LDL receptor is an endocytic receptor that transports lipoproteins, mainly the cholesterol-rich lipoprotein LDL, into cells through receptor-mediated endocytosis [26,27]. This process involves the cell surface receptor recognizing an LDL particle, followed by its internalization through clathrin-coated pits [28,29]. It has been suggested that HCV might enter the cells via the LDL receptor [8,9]. The binding of low density HCV particles correlated with the extent of the LDL receptor at the cell surface, but not soluble CD81 [30]. In contrast, the binding of HCV-like particles did not correlate with the LDL receptor expression, but was CD81-independent. These hepatoma and lymphoma cells were directly incubated with the virus-like particles [19], without previous removal of the cell-bound lipoproteins. Moreover, free beta-lipoproteins in human serum may influence the rate of infection of hepatocytes by competing with the virus. In support of this, it has been shown that the LDL receptor can function as a HCV receptor and that beta-lipoproteins competitively inhibit the infection of hepatocytes with HCV through the LDL receptor [31]. Indeed, it has been suggested that the removal of the cell-bound lipoproteins is a crucial prerequisite for the infection of hepatocytes with HCV [32]. In the latter study, the viral inoculum that was employed was composed of a virus-lipoprotein complex, as described elsewhere [33,34].

### HCV and exosomes

Exosomes are small membrane vesicles secreted by cells upon fusion of multivesicular endosomes with the cell surface [35,36]. They are 60 to 100 nm in diameter and originate from late endosomes. Exosomes are secreted from most hematopoietic and epithelial cells in vitro. Intracellularly, they are formed by inward budding of the endosomal membrane in a process that sequesters particular proteins and lipids [37]. The unique composition of exosomes may confer specific functions to them upon secretion.

Recently, it has been shown that the HCV envelope proteins were associated with exosomes [38]. In the absence of the human CD81, HCV envelope proteins were almost completely retained in the endoplasmic reticulum of hamster CHO cells. Instead, when the human CD81 was present, a fraction of the HCV envelope proteins passed through the Golgi apparatus, matured acquiring complex sugars and was found extracellularly associated with exosomes. It was proposed that the HCV-CD81 complex exits the cells in the form of exosomes, circulates in the blood as a complex and exploits the fusogenic capabilities of these vesicles to infect cells even in the presence of neutralizing antibodies. Therefore, the human CD81 may in fact act as an exit receptor for HCV. The authors concluded that a fraction of HCV RNA was bound to CD81 in patients infected with HCV, because it was difficult to estimate the exact fraction of HCV RNA in human plasma that was associated with exosomes. Differential centrifugation was employed for the purification of the exosomes, so that the buoyant density of these vesicles was not measured. This measure would be important to discriminate between free and bound HCV [38].

Lipid raft-associated protein sorting has been involved with exosomes. Some molecules are released in the extracellular medium via their association with lipid raft domains of the exosomal membrane. The presence of lipid microdomains in exosomal membranes is suggesting their participation in vesicle formation and structure, as well as the direct implication of exosomes in regulatory mechanisms [39]. Buoyant density is the quality for a compound to rise or float in a liquid. The measure of this density can be employed for the discrimination of exosomes, for example. Exosomes float to a density close to 1.13 g/ml (as revealed by ultracentrifugation), but this may vary from cell to cell depending of the exosome protein content [37,40]. In several studies, the buoyant density of exosomes originating from B lymphocytes has been determined to be in the range 1.08–1,22 g/ml with a peak at 1.13–1.15 g/ml [41,42].

Interestingly, hepatitis C virus is structurally migrating with buoyant densities lying in the same range as those determined for low density lipoproteins and exosomes. It was shown that HCV was associated with beta-lipoproteins having buoyant densities between 1.03 g/ml and 1.20 g/ml in the human serum [33,34]. Moreover, action of lipoprotein lipase on hepatitis C virus in human sera was shown to be virolytic [43]. In order to analyze the potential HCV-lipoprotein complex, the binding of sucrose gradient-purified low-density particles (1.03 to 1.07 g/cm^3^), intermediate-density particles (1.12 to 1.18 g/cm^3^), recombinant E2 protein, or control proteins to MOLT-4 cells, foreskin fibroblasts, or LDL receptor-deficient foreskin fibroblasts, was assessed. This revealed that the low-density HCV particles, but not intermediate-density HCV or controls, bound to MOLT-4 cells and fibroblasts expressing the LDL receptor. Binding correlated with the extent of cellular LDL receptor expression and was inhibited by LDL but not by soluble CD81. In contrast, E2 binding was independent of LDL receptor expression and was inhibited by human soluble CD81 but not mouse soluble CD81 or LDL [30].

The complete complementary DNA of an isolate of the hepatitis C virus was cloned into a tetracycline-inducible expression vector and stably transfected into the human hepatoma cell lines Huh7 and HepG2. Viral RNA levels peaked at two separate ranges, one with a buoyant density of 1.08 g/ml and another from 1.17 to 1.39 g/ml. Anti-E2 antibodies strongly labelled cytoplasmic vesicular structures and some viral-like particles. Complete viral particles of about 50 nm which reacted with anti-E2 antibodies were observed in the culture media of tet-induced clonal HuH-7 cells following negative staining [44].

### A general scheme for the adsorption and penetration of HCV onto cells is emerging

It is tempting to speculate that HCV bound to, or contained within, low density lipoproteins [33,34], viro-lipo-particles [45], or exosomes [38] would be constituted as one similar structure that would allow the virus to adsorb and penetrate into the target cells. This structure would in general migrate in sucrose gradients at a density similar to low density lipoproteins. A common feature of these three different structures is that they all contain lipids such as sphingomyelin, cholesterol, glycolipids and lipids that are critical for the maintenance of lipid rafts [37,40]. Consistent with the initial buoyant density measures [33,34], the lipoproteins bound to HCV might therefore be similar to those present in exosomes [38]. Since hepatocytes are from hematopoïetic origin [46], they are producing exosomes [47]. Moreover, since CD81 is enriched in exosomes [48], it may not be a receptor mediating the HCV entry into hepatocytes, but rather an exit receptor. Although SR-BI is highly expressed in hepatocytes [22,25] and is a receptor for HDL and LDL [22,49], hepatocyte cell lines such as HepG2 do not express CD81 [7]. Therefore, the afforementioned compilation of observations might argue against the role played by CD81 as the cellular receptor for HCV. Therefore, since low density lipoproteins and exosomes do bind the LDL receptor [50], HCV might enter the cells via the LDL receptor solely. Work is currently under progress in order to analyze the uptake of the hepatitis C virus-lipoprotein in HepG2 and HuH-7 cells in absence or presence of N^4^-octadecyl-1-β-D-arabinofuranosylcytosine, as described elsewhere [51].

## Conclusion

### In vitro and in vivo implications

**Figure 1:**
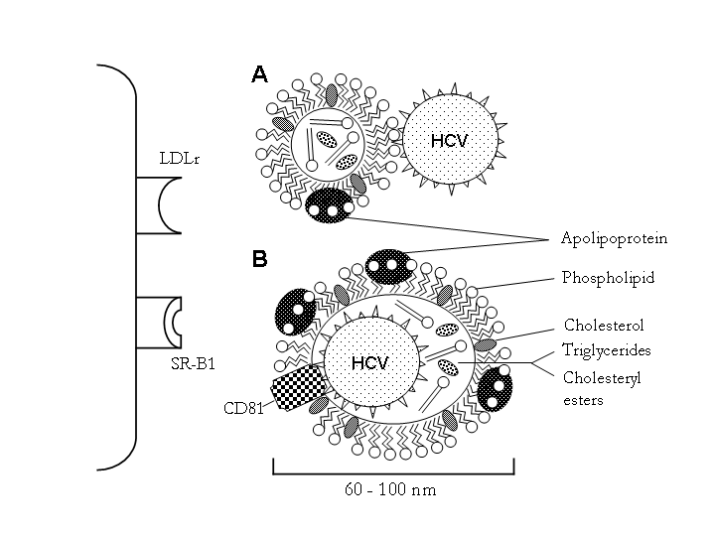
A model for HCV infection. **A. **HCV is present as a virus-lipoprotein complex in the human blood. HCV can be surrounded by several low density lipoproteins, so that the viral envelope proteins E1 and E2 might be masked. **B. **HCV is present within exosomes. It was recently shown that these exosomes are containing, apart from the genomic ribonucleic acid of HCV, the tetraspanin CD81 associated with E1 and E2. It is likely that the binding of the HCV-lipoprotein and/or the exosome-HCV complexes to the low density lipoprotein receptor (LDLr) might therefore be hampered in vitro by the cell-bound lipoproteins, or by the vast excess of free lipoproteins in the human blood. Therefore, it may be beneficial to remove the cell-bound lipoproteins with dextran sulfate (thus generating free LDL receptors) prior to the addition of the viral inoculum onto the target hepatocytes for the generation of an in vitro infection. The same may be true for the scavenger receptor SR-BI, since it does also bind LDL. In vivo, the use of statins may enhance the rate of HCV infection in HCV-infected patients, because of the increase of the LDL receptors at the surface of the hepatocytes.

It might be envisioned that one of the crucial parameter for viral entry would therefore be the LDL receptor at the cell surface of the hepatocytes. If this is true, the CD81 tetraspanin and/or SR-B1 would rather regulate virus adhesion and/or fusion with target cells than playing the role of a cellular receptor (Fig. 1). Indeed, it has been proposed that in order to infect hepatocyte cell lines with HCV in vitro, the cell-bound lipoproteins have to be removed with dextran sulfate prior to the addition of the viral inoculum onto the cells [32]. According to this model, the binding of the HCV-lipoprotein complex to the LDL receptor might be hampered in vitro by the cell-bound lipoproteins, or by the vast excess of free lipoproteins over virus-bound lipoproteins in the human blood. The similar procedure of infection would therefore also apply for viro-lipo-particles and exosomes.

The lipidemic status in HCV-infected individuals might also play a critical role for the onset and the maintainance of a robust immune response directed against HCV, especially in patients suffering from hypercholesterolemia, coronary artery disease, diabetes mellitus or obesity. Cholesterol lowering drugs with statins are abundantly employed for the lipid management in hyperlipidemic patients [52,53]. The primary effect of statins is the induction of the expression of LDL receptors on the surface of the hepatocytes [54,55]. Safety and tolerability profiles are available and statins have become the drugs of choice when diet alone has failed [56]. However, there are no controlled trials published that might reveal the link between the cholesterol management with statins and the efficiency of the hepatitis C virus replication, yet. It remains obviously to be shown, whether the recovery from hyperlipidemia to normo- or hypolipidemia is indeed not dramatically allowing the infection or the re-infection of hepatocytes with HCV in the human liver.

## Authors' contributions

DF wrote the initial draft of the article and created the figure 1. BM critically revised the article until its final version. Both authors read and approved the final manuscript.

## References

[B1] Lauer GM, Walker BD (2001). Hepatitis C virus infection. N Engl J Med.

[B2] Koike K, Tsutsumi T, Fujie H, Shintani Y, Kyoji M (2002). Molecular mechanism of viral hepatocarcinogenesis. Oncology.

[B3] Moriishi K, Matsuura Y (2003). Mechanisms of hepatitis C virus infection. Antivir Chem Chemother.

[B4] Nishioka T, Chayama K (2004). [Mechanism of HCV cell entry mediated by envelope and receptor proteins]. Nippon Rinsho.

[B5] Flint M, Maidens C, Loomis-Price LD, Shotton C, Dubuisson J, Monk P, Higginbottom A, Levy S, McKeating JA (1999). Characterization of hepatitis C virus E2 glycoprotein interaction with a putative cellular receptor, CD81. J Virol.

[B6] Pileri P, Uematsu Y, Campagnoli S, Galli G, Falugi F, Petracca R, Weiner AJ, Houghton M, Rosa D, Grandi G (1998). Binding of hepatitis C virus to CD81. Science.

[B7] Scarselli E, Ansuini H, Cerino R, Roccasecca RM, Acali S, Filocamo G, Traboni C, Nicosia A, Cortese R, Vitelli A (2002). The human scavenger receptor class B type I is a novel candidate receptor for the hepatitis C virus. Embo J.

[B8] Monazahian M, Bohme I, Bonk S, Koch A, Scholz C, Grethe S, Thomssen R (1999). Low density lipoprotein receptor as a candidate receptor for hepatitis C virus. J Med Virol.

[B9] Agnello V, Abel G, Elfahal M, Knight GB, Zhang QX (1999). Hepatitis C virus and other flaviviridae viruses enter cells via low density lipoprotein receptor. Proc Natl Acad Sci U S A.

[B10] Levy S, Todd SC, Maecker HT (1998). CD81 (TAPA-1): a molecule involved in signal transduction and cell adhesion in the immune system. Annu Rev Immunol.

[B11] Cormier EG, Tsamis F, Kajumo F, Durso RJ, Gardner JP, Dragic T (2004). CD81 is an entry coreceptor for hepatitis C virus. Proc Natl Acad Sci U S A.

[B12] Lambot M, Fretier S, Op De Beeck A, Quatannens B, Lestavel S, Clavey V, Dubuisson J (2002). Reconstitution of hepatitis C virus envelope glycoproteins into liposomes as a surrogate model to study virus attachment. J Biol Chem.

[B13] Higginbottom A, Quinn ER, Kuo CC, Flint M, Wilson LH, Bianchi E, Nicosia A, Monk PN, McKeating JA, Levy S (2000). Identification of amino acid residues in CD81 critical for interaction with hepatitis C virus envelope glycoprotein E2. J Virol.

[B14] Hsu M, Zhang J, Flint M, Logvinoff C, Cheng-Mayer C, Rice CM, McKeating JA (2003). Hepatitis C virus glycoproteins mediate pH-dependent cell entry of pseudotyped retroviral particles. Proc Natl Acad Sci U S A.

[B15] Tan YJ, Lim SP, Ng P, Goh PY, Lim SG, Tan YH, Hong W (2003). CD81 engineered with endocytotic signals mediates HCV cell entry: implications for receptor usage by HCV in vivo. Virology.

[B16] Zhang J, Randall G, Higginbottom A, Monk P, Rice CM, McKeating JA (2004). CD81 is required for hepatitis C virus glycoprotein-mediated viral infection. J Virol.

[B17] Petracca R, Falugi F, Galli G, Norais N, Rosa D, Campagnoli S, Burgio V, Di Stasio E, Giardina B, Houghton M (2000). Structure-function analysis of hepatitis C virus envelope-CD81 binding. J Virol.

[B18] Meola A, Sbardellati A, Bruni Ercole B, Cerretani M, Pezzanera M, Ceccacci A, Vitelli A, Levy S, Nicosia A, Traboni C (2000). Binding of hepatitis C virus E2 glycoprotein to CD81 does not correlate with species permissiveness to infection. J Virol.

[B19] Wellnitz S, Klumpp B, Barth H, Ito S, Depla E, Dubuisson J, Blum HE, Baumert TF (2002). Binding of hepatitis C virus-like particles derived from infectious clone H77C to defined human cell lines. J Virol.

[B20] Masciopinto F, Freer G, Burgio VL, Levy S, Galli-Stampino L, Bendinelli M, Houghton M, Abrignani S, Uematsu Y (2002). Expression of human CD81 in transgenic mice does not confer susceptibility to hepatitis C virus infection. Virology.

[B21] Krieger M, Stern DM (2001). Series introduction: multiligand receptors and human disease. J Clin Invest.

[B22] Krieger M (2001). Scavenger receptor class B type I is a multiligand HDL receptor that influences diverse physiologic systems. J Clin Invest.

[B23] Acton S, Rigotti A, Landschulz KT, Xu S, Hobbs HH, Krieger M (1996). Identification of scavenger receptor SR-BI as a high density lipoprotein receptor. Science.

[B24] Calvo D, Gomez-Coronado D, Lasuncion MA, Vega MA (1997). CLA-1 is an 85-kD plasma membrane glycoprotein that acts as a high-affinity receptor for both native (HDL, LDL, and VLDL) and modified (OxLDL and AcLDL) lipoproteins. Arterioscler Thromb Vasc Biol.

[B25] Calvo M, Enrich C (2000). Biochemical analysis of a caveolae-enriched plasma membrane fraction from rat liver. Electrophoresis.

[B26] Chung NS, Wasan KM (2004). Potential role of the low-density lipoprotein receptor family as mediators of cellular drug uptake. Adv Drug Deliv Rev.

[B27] Nykjaer A, Willnow TE (2002). The low-density lipoprotein receptor gene family: a cellular Swiss army knife?. Trends Cell Biol.

[B28] Steer CJ, Zakim D, Boyer TD (1996). Receptor-mediated endocytosis: mechanisms, biologic function, and molecular properties. Hepatology: a textbook of liver disease.

[B29] Cooper AD, Ellsworth JL, Zakim D, Boyer TD (1996). Lipoprotein metabolism. Hepatology: a textbook of liver disease.

[B30] Wunschmann S, Medh JD, Klinzmann D, Schmidt WN, Stapleton JT (2000). Characterization of hepatitis C virus (HCV) and HCV E2 interactions with CD81 and the low-density lipoprotein receptor. J Virol.

[B31] Enjoji M, Nakamuta M, Kinukawa N, Sugimoto R, Noguchi K, Tsuruta S, Iwao M, Kotoh K, Iwamoto H, Nawata H (2000). Beta-lipoproteins influence the serum level of hepatitis C virus. Medical Science Monitor.

[B32] Favre D, Berthillon P, Trepo C (2001). Removal of cell-bound lipoproteins: a crucial step for the efficient infection of liver cells with hepatitis C virus in vitro. C R Acad Sci III.

[B33] Thomssen R, Bonk S, Propfe C, Heermann KH, Kochel HG, Uy A (1992). Association of hepatitis C virus in human sera with beta-lipoprotein. Med Microbiol Immunol (Berl).

[B34] Thomssen R, Bonk S, Thiele A (1993). Density heterogeneities of hepatitis C virus in human sera due to the binding of beta-lipoproteins and immunoglobulins. Med Microbiol Immunol (Berl).

[B35] Farsad K (2002). Exosomes: novel organelles implicated in immunomodulation and apoptosis. Yale J Biol Med.

[B36] Stahl PD, Barbieri MA (2002). Multivesicular bodies and multivesicular endosomes: the "ins and outs" of endosomal traffic. Sci STKE.

[B37] Stoorvogel W, Kleijmeer MJ, Geuze HJ, Raposo G (2002). The biogenesis and functions of exosomes. Traffic.

[B38] Masciopinto F, Giovani C, Campagnoli S, Galli-Stampino L, Colombatto P, Brunetto M, Yen TS, Houghton M, Pileri P, Abrignani S (2004). Association of hepatitis C virus envelope proteins with exosomes. Eur J Immunol.

[B39] de Gassart A, Geminard C, Fevrier B, Raposo G, Vidal M (2003). Lipid raft-associated protein sorting in exosomes. Blood.

[B40] Fevrier B, Raposo G (2004). Exosomes: endosomal-derived vesicles shipping extracellular messages. Curr Opin Cell Biol.

[B41] Raposo G, Nijman HW, Stoorvogel W, Liejendekker R, Harding CV, Melief CJ, Geuze HJ (1996). B lymphocytes secrete antigen-presenting vesicles. J Exp Med.

[B42] Wubbolts R, Leckie RS, Veenhuizen PT, Schwarzmann G, Mobius W, Hoernschemeyer J, Slot JW, Geuze HJ, Stoorvogel W (2003). Proteomic and biochemical analyses of human B cell-derived exosomes. Potential implications for their function and multivesicular body formation. J Biol Chem.

[B43] Thomssen R, Bonk S (2002). Virolytic action of lipoprotein lipase on hepatitis C virus in human sera. Med Microbiol Immunol (Berl).

[B44] Lim SP, Soo HM, Tan YH, Brenner S, Horstmann H, MacKenzie JM, Ng ML, Lim SG, Hong W (2002). Inducible system in human hepatoma cell lines for hepatitis C virus production. Virology.

[B45] Andre P, Komurian-Pradel F, Deforges S, Perret M, Berland JL, Sodoyer M, Pol S, Brechot C, Paranhos-Baccala G, Lotteau V (2002). Characterization of low- and very-low-density hepatitis C virus RNA-containing particles. J Virol.

[B46] Lagasse E, Connors H, Al-Dhalimy M, Reitsma M, Dohse M, Osborne L, Wang X, Finegold M, Weissman IL, Grompe M (2000). Purified hematopoietic stem cells can differentiate into hepatocytes in vivo. Nat Med.

[B47] Denzer K, Kleijmeer MJ, Heijnen HF, Stoorvogel W, Geuze HJ (2000). Exosome: from internal vesicle of the multivesicular body to intercellular signaling device. J Cell Sci.

[B48] Escola JM, Kleijmeer MJ, Stoorvogel W, Griffith JM, Yoshie O, Geuze HJ (1998). Selective enrichment of tetraspan proteins on the internal vesicles of multivesicular endosomes and on exosomes secreted by human B-lymphocytes. J Biol Chem.

[B49] Krieger M (1999). Charting the fate of the "good cholesterol": identification and characterization of the high-density lipoprotein receptor SR-BI. Annu Rev Biochem.

[B50] Laulagnier K, Motta C, Hamdi S, Roy S, Fauvelle F, Pageaux JF, Kobayashi T, Salles JP, Perret B, Bonnerot C (2004). Mast cell- and dendritic cell-derived exosomes display a specific lipid composition and an unusual membrane organization. Biochem J.

[B51] Koller-Lucae SK, Schott H, Schwendener RA (1999). Low density lipoprotein and liposome mediated uptake and cytotoxic effect of N4-octadecyl-1-beta-D-arabinofuranosylcytosine in Daudi lymphoma cells. Br J Cancer.

[B52] McKenney JM (2001). Lipid management: tools for getting to the goal. Am J Manag Care.

[B53] Stein EA (1989). Management of hypercholesterolemia. Approach to diet and drug therapy. Am J Med.

[B54] Ginsberg HN (1998). Effects of statins on triglyceride metabolism. Am J Cardiol.

[B55] Duriez P (2003). [Mechanisms of actions of statins and fibrates]. Therapie.

[B56] Jenkins DJ, Kendall CW, Marchie A, Faulkner D, Vidgen E, Lapsley KG, Trautwein EA, Parker TL, Josse RG, Leiter LA (2003). The effect of combining plant sterols, soy protein, viscous fibers, and almonds in treating hypercholesterolemia. Metabolism.

